# The ATP Receptors P2X7 and P2X4 Modulate High Glucose and Palmitate-Induced Inflammatory Responses in Endothelial Cells

**DOI:** 10.1371/journal.pone.0125111

**Published:** 2015-05-04

**Authors:** Ramasri Sathanoori, Karl Swärd, Björn Olde, David Erlinge

**Affiliations:** 1 Department of Cardiology, Clinical Sciences, Lund University, Lund, Sweden; 2 Department of Experimental Medical Science, Lund University, Lund, Sweden; Universidad Autonoma de San Luis Potosi, MEXICO

## Abstract

Endothelial cells lining the blood vessels are principal players in vascular inflammatory responses. Dysregulation of endothelial cell function caused by hyperglycemia, dyslipidemia, and hyperinsulinemia often result in impaired vasoregulation, oxidative stress, inflammation, and altered barrier function. Various stressors including high glucose stimulate the release of nucleotides thus initiating signaling via purinergic receptors. However, purinergic modulation of inflammatory responses in endothelial cells caused by high glucose and palmitate remains unclear. In the present study, we investigated whether the effect of high glucose and palmitate is mediated by P2X7 and P2X4 and if they play a role in endothelial cell dysfunction. Transcript and protein levels of inflammatory genes as well as reactive oxygen species production, endothelial-leukocyte adhesion, and cell permeability were investigated in human umbilical vein endothelial cells exposed to high glucose and palmitate. We report high glucose and palmitate to increase levels of extracellular ATP, expression of P2X7 and P2X4, and inflammatory markers. Both P2X7 and P2X4 antagonists inhibited high glucose and palmitate-induced interleukin-6 levels with the former having a significant effect on interleukin-8 and cyclooxygenase-2. The effect of the antagonists was confirmed with siRNA knockdown of the receptors. In addition, P2X7 mediated both high glucose and palmitate-induced increase in reactive oxygen species levels and decrease in endothelial nitric oxide synthase. Blocking P2X7 inhibited high glucose and palmitate-induced expression of intercellular adhesion molecule-1 and vascular cell adhesion molecule-1 as well as leukocyte-endothelial cell adhesion. Interestingly, high glucose and palmitate enhanced endothelial cell permeability that was dependent on both P2X7 and P2X4. Furthermore, antagonizing the P2X7 inhibited high glucose and palmitate-mediated activation of p38-mitogen activated protein kinase. These findings support a novel role for P2X7 and P2X4 coupled to induction of inflammatory molecules in modulating high glucose and palmitate-induced endothelial cell activation and dysfunction.

## Introduction

The metabolic syndrome is a condition that is defined by increased insulin resistance, type 2 diabetes (T2D), obesity, and hypertension. High levels of glucose and circulating free fatty acids are important in the etiology of this condition usually associated with chronic low-grade inflammation evident both systemically and locally in the affected tissues [1–4]. Oxidative stress and inflammation give rise to vascular endothelial dysfunction, which is an early event in the development of atherosclerosis [1,4,5].

The vascular endothelium is a source of autocrine and paracrine mediators that modulate vascular tone, cell adhesion, permeability, and vessel wall inflammation wherein stimuli such as extracellular adenosine 5’-triphosphate (eATP), glucose, and palmitate are known triggers for inflammatory responses or activation of the inflammasome in various cell types [6–9]. A consequence of endothelial cell damage and lysis at the site of inflammation is increased ATP in the extracellular space [10–12]. Moreover, high glucose is known to release eATP, which can orchestrate a cascade of events that lead to activation of the purinergic-signaling axis [13,14]. Increasing evidence points to the role of P2X7 and P2X4 in modulating inflammatory responses in both immune and non-immune cells as well as in the retinal and renal microvasculature [15–20]. In fact, alterations in P2X7 expression and receptor-dependent responses are reported in T2D patient fibroblasts, β cells, and peripheral blood mononuclear cells (PBMCs) implicating this receptor in T2D pathogenesis [21–23].

Endothelial dysfunction is an important factor in the progression of diabetes-associated cardiovascular complications. It is known that elevated production of free fatty acids can enhance vascular insulin resistance by inhibiting insulin signaling [24–26]. Not only do high glucose, excess fatty acids, and insulin resistance individually enhance the development and progression of endothelial dysfunction, but also the combined effect of them can potentially aggravate this condition. In spite of several reports demonstrating pathogenic effects of either excess glucose or palmitate on endothelial cells [6–9,27,28], little is known about the role of purinergic receptors in mediating these effects. We therefore investigated the high glucose and palmitate-mediated inflammatory response in human umbilical vein endothelial cells (HUVECs) with a focus on the role of P2X7 and P2X4. We report differential effects of these P2X receptors in modulating the inflammatory effects of high glucose and palmitate in endothelial cell dysfunction.

## Materials and Methods

### Reagents

Medium 200, basic fibroblast growth factor / heparin, hydrocortisone, human epidermal growth factor, gentamycin / amphotericin, fetal bovine serum (FBS), 1X attachment factor, High capacity cDNA reverse transcription kit, TaqMan assays and master mixes, Lipofectamine RNAiMAX transfection reagent, OPTI-MEM, Silencer Select siRNA for P2X7 and P2X4, XT 4–12% Bis-Tris gels, and ProLong gold antifade mounting medium were all obtained from Life Technologies, USA. QIAzol lysis reagent, miRNeasy mini kit and RNase free DNase set were from Qiagen, USA. Sodium palmitate, D-Glucose, Fatty acid-free bovine serum albumin (BSA), adenosine 5’-[γ-thio]triphosphate tetralithium salt (ATPγS), 2’(3’)-O-(4-Benzoylbenzoyl)-adenosine 5’-triphosphate triethylammonium salt (BzATP), apyrase, fluorescein thiocyanate (FITC)-Dextran40, and dimethyl sulfoxide (DMSO) were from Sigma, USA; ATP SL kit from Biothema, Sweden; P2X7 antagonists AZ11645373 and A438079 from TOCRIS, UK; Phospho-Stop and complete protease inhibitors from Roche, USA; Quantikine enzyme-linked immunosorbent assays (ELISA) for interleukin(IL)-6 and IL-8 (R&D Systems, USA); Micro bicinchoninic acid assay (BCA) protein assay kit from Thermo Fisher, Sweden, and the P2X4 antagonist, PSB-12253, was a kind gift from Prof. Christa Müller (University of Bonn, Germany). All other reagents were purchased from Sigma unless otherwise mentioned. The following antibodies were used: vascular cell adhesion molecule-1 (VCAM-1) (Millipore & Cell Signaling Technology, USA), intercellular adhesion molecule-1 (ICAM-1) (Cell Signaling Technology), and Alexa Fluor 488 and Alexa Fluor 568 secondary antibodies (Life Technologies), and NucBlue (Molecular Probes, USA), P2X4 and P2X7 antibodies and control antigens (Alomone Labs, Israel), β-tubulin, cyclooxygenase-2 (COX-2), phosphorylated and total p38-mitogen activated protein kinase (p38-MAPK) (Cell Signaling Technology).

### Cell culture

Pooled HUVECs from multiple donors (Invitrogen, USA) were used between passages 1–4. The cells were routinely cultured in normal medium 200 supplemented with basic fibroblast growth factor / heparin, hydrocortisone, human epidermal growth factor, gentamycin / amphotericin, and 10% fetal bovine serum (FBS). The cells were exposed to high glucose and palmitate (final concentration of 30 mmol/L glucose and 150 μmol/L palmitate conjugated to fatty acid-free BSA) in normal growth medium containing 1% serum for 24 and/or 48 h. Cells were incubated in the presence or absence of 5 U/ml apyrase, 100 nmol/L of AZ11645373, and 50 μmol/L A438079, (P2X7 antagonists) and 1 μmol/L of PSB-12253 (P2X4 antagonist), which were all added 1 h prior to subjecting the cells to high glucose and palmitate.

### siRNA-mediated knockdown

HUVECs were seeded to achieve a confluence of 70–80% on the day of transfection. Transient transfection with P2X4 and P2X7-specific siRNA was performed as previously described [29]. In brief, siRNA at a final concentration of 10 nM/L was incubated with Lipofectamine RNAiMAX reagent in OPTI-MEM to form complexes. At 48 h post-transfection, the cells were exposed to high glucose and palmitate for 24 h and harvested for RNA.

### Luminometric measurement of eATP

Confluent HUVECs cultured in 12-well plates were exposed to high glucose and palmitate and supernatants were collected at time zero, 24 h, and 48 h. The ATP in the supernatants was measured using the ATP reagent SL and an internal ATP standard (BioThema, Sweden) as per the manufacturer’s protocol. Briefly, the luciferase activity in the supernatants was measured with the GloMax 20/20 Luminometer (Promega, USA).

### RNA isolation and reverse transcription-PCR analysis

Cells were harvested and lysed with QIAzol and total RNA was extracted using the miRNeasy mini kit with the DNAse I step to remove contaminating DNA. First-strand cDNA was synthesized from 1 μg of RNA using the high capacity cDNA reverse transcription kit. Negative controls with no template and no reverse transcriptase enzyme were also included. Quantitative real-time PCR (qRT-PCR) was performed using the ABI StepOnePlus instrument as per manufacturer’s instructions. TaqMan assays were used to measure mRNA expression, which were normalized using the housekeeping genes *PPIA*, and *18S*.

### IL-6 and IL-8 ELISAs

Secreted IL-6 and IL-8 in the supernatants of HUVECs exposed to 48 h high glucose and palmitate in the presence or absence of antagonists was quantified using the colorimetric human IL-6 and IL-8 ELISA kits as per the manufacturer’s protocol. The absorbance was measured at 450 nm with a correction wavelength set at 570 nm and the concentrations were determined by interpolation from a standard calibration curve.

### Immunoblot analysis

For immunoblotting, protein was extracted from cells using ice-cold Nonidet P-40 lysis buffer (50 mmol/L Tris(hydroxymethyl)aminomethane hydrochloride (Tris-HCl) (pH 7.5), 1 mmol/L ethylene glycol tetraacetic acid (EGTA), 1 mmol/L ethylenediaminetetraacetic acid (EDTA), 1 mmol/L sodium orthovanadate, 10 mmol/L sodium-β-glycerophosphate, 50 mmol/L sodium fluoride, 5 mmol/L sodium pyrophosphate, 1 mmol/L dithiothreitol (DTT), 1% (w/v) Nonidet P-40, and 270 mmol/L sucrose) with complete protease and phosphatase inhibitor mixture. Lysates were centrifuged at 14,000xg for 15 min at 4°C, and the protein concentration was determined using Micro-BCA protein assay kit. The total cell lysates were heated at 95°C for 5 min in lauryl dodecyl sulfate sample buffer. Total protein (10–15 μg) was resolved on precast NOVEX 4–12% Bis-Tris gels and transferred onto nitrocellulose membranes. The membranes were blocked for 1 h at room temperature in 50 mmol/L Tris-HCl (pH 7.6), 137 mmol/L sodium chloride (NaCl), and 0.2% (w/v) Tween 20 (TBS-T) containing either 5% (w/v) nonfat dry milk or 3% (w/v) protease-free BSA followed by overnight incubation at 4°C with the indicated antibodies (1:500 to 1:1000) in TBS-T containing 5% (w/v) protease-free BSA. The bands were visualized by enhanced chemiluminescence using horseradish peroxidase-conjugated secondary antibody (1:2500). Images were acquired with LI-COR Odyssey Fc dual-mode imaging system and the band intensities were quantified using the Image Studio software. To test the specificity of P2X7, the anti-P2X7 antibody was pre-adsorbed using the control antigen. Phosphorylated proteins were normalized to the respective total protein. Other proteins were normalized to β-Tubulin and all changes in protein levels are depicted as relative to the control condition.

### Intracellular reactive oxygen species (ROS) measurement

The fluorescent probe 6-carboxy-2',7'-dichlorodihydrofluorescein diacetate (H_2_DCFDA) prepared in 1X Hank’s balanced salt solution (HBSS) at a concentration of 10 μmol/L was used to assess intracellular ROS. HUVECs seeded in black 96-well plates were exposed to high glucose and palmitate in the presence or absence of the antagonists. The cells were then washed once with 1X HBSS, followed by the addition of H_2_DCFDA, and incubation of the cells at 37°C for 30 min. Subsequently the dye was aspirated from the wells, pre-warmed growth medium added, and the cells returned to the incubator to recover for an additional hour. The fluorescence was recorded as a single endpoint measurement at excitation and emission wavelengths of 485 nm and 530 nm, respectively.

### Immunocytochemistry

HUVEC cultures after exposure to high glucose and palmitate were washed with 1X phosphate buffered saline (PBS) and fixed with ice-cold 4% paraformaldehyde for 20 min at room temperature. They were then washed once and permeabilized with 0.25% Triton-X100 in 1X PBS for 15 min at room temperature. Subsequent to washes, they were incubated in the primary antibody overnight at 4°C in a humidified chamber. The following day, the cells were washed 3 times in 1X PBS and incubated in the Alexa Fluor 488 or Alexa Fluor 568 secondary antibodies for 2 h at room temperature in the dark. Finally, the cells were stained with NucBlue ready probes as per the manufacturer’s protocol. The cells on coverslips were mounted with ProLong gold antifade. Images were visualized in an Olympus BX60 (Tokyo, Japan) epifluorescence microscope, and acquired using a Nikon DS-2Mv (Tokyo, Japan).

### Leukocyte preparation and Leukocyte-endothelium Adhesion Assay

Citrated whole blood (50 ml) was drawn from healthy adult volunteers with their informed written consent and according to the principles of The Declaration of Helsinki. The ethics committee of the Faculty of Medicine at Lund University approved this study. PBMCs were isolated using Lymphoprep (Axis-Shield, Norway), washed twice with 1X PBS, re-suspended at 1x10^6^ cells/ml in serum free media, and labeled with the LeukoTracker (Cell Biolabs Inc., USA) as per the manufacturer’s protocol.

HUVECs were seeded at 5-10x10^4^ cells per well in 1X attachment factor-coated 96-well plates and cultured for 48 h until they form a monolayer. The cells were subsequently exposed to high glucose and palmitate in the presence or absence of the P2X7 and P2X4 antagonists for 48 h. The labeled leukocytes were added to the endothelial monolayer and incubated for 90 min at 37°C / 5% CO_2_. The media was then aspirated from each well, followed by three washes and cell lysis. The fluorescence was then measured at excitation and emission wavelengths of 480 nm and 520 nm, respectively.

### FITC-dextran permeability Assay

HUVEC monolayer leakage was measured using the transwell permeable inserts (0.4 μm) and FITC-dextran (MW 40,000 Da). Briefly, the transwell membrane insert was coated with 1X attachment factor and HUVECs were seeded at 1x10^5^ cells per insert in a final volume of 100 μl of complete growth medium. The inserts were placed in 24-well plates containing 600 μl of growth medium and cultured for 48 h before exposure to antagonists and high glucose and palmitate. HUVECs were exposed to the antagonists for 1 h followed by the addition of high glucose and palmitate for 48 h. Subsequently, the culture medium was removed and the basal media without phenol red was added to both the inserts and wells. FITC-dextran (1 mg/ml) was added to the inserts and incubated at 37°C / 5% CO_2_ for 1 h. The insert was then removed and 100 μl of medium collected from the lower chamber. The fluorescence was measured at excitation and emission wavelengths of 485 nm and 530 nm respectively. Endothelial cell permeability was evaluated in controls and experimental conditions performed in replicates of 2 to 3 wells.

### Statistical analysis

All data are expressed as mean ± standard error (s.e.m.). Statistical analysis (GraphPad Prism Inc., USA) was performed using unpaired Student’s *t*-test when comparing 2 situations, or one-way analysis of variance (ANOVA) with Bonferroni correction for multiple comparisons with *p* values ≤ 0.05 regarded as statistically significant.

## Results

### Effect of high glucose and palmitate on endothelial gene expression associated with inflammation

**Fig 1:**
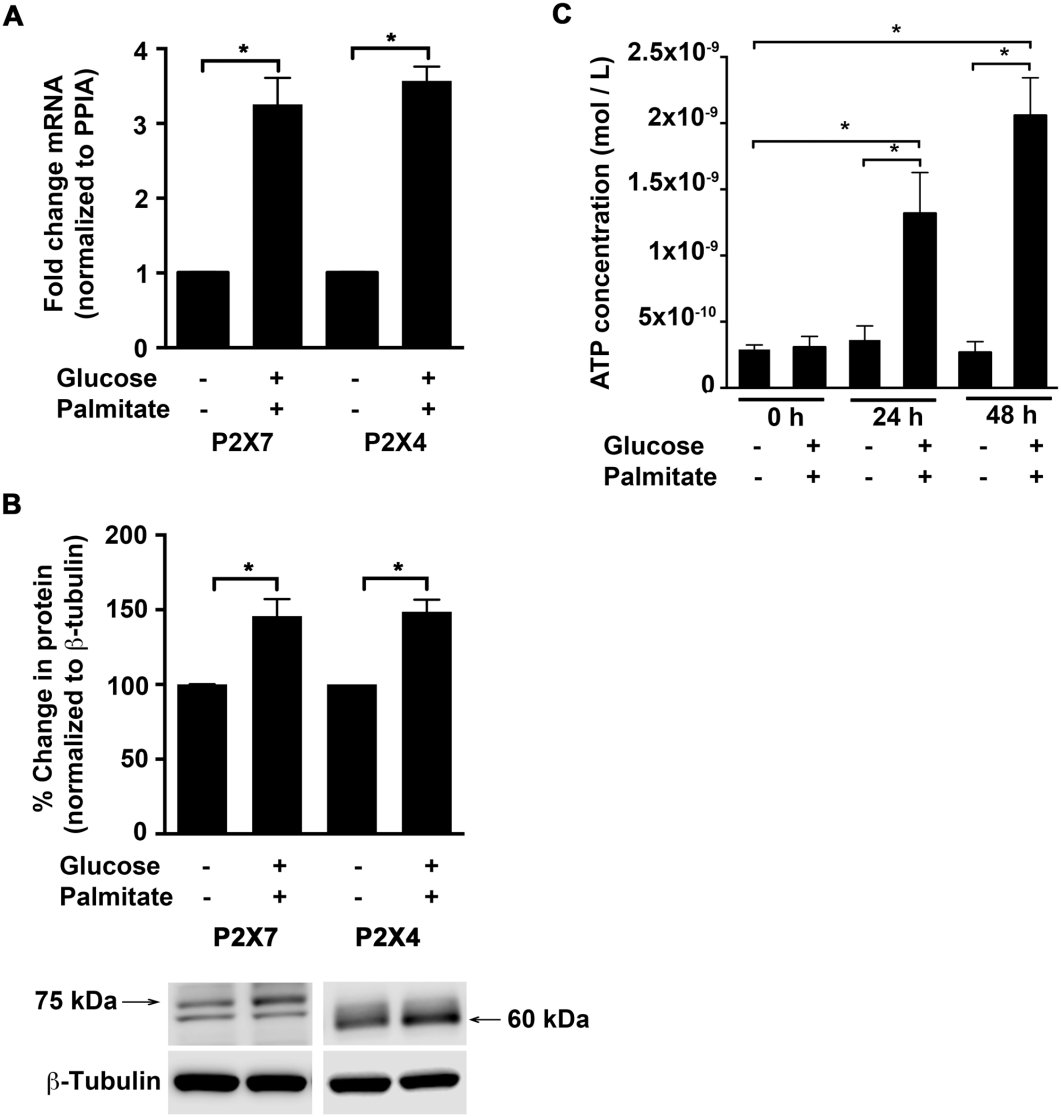
High glucose and palmitate increases P2X7 and P2X4 expression and eATP release. qRT-PCR analysis (A) shows high glucose and palmitate (24 h) to induce *P2X7* and *P2X4* transcript levels normalized to the housekeeping gene, *PPIA*. Immunoblot analysis (B) shows protein levels of P2X7 and P2X4 in response to high glucose and palmitate (48 h) normalized to β-Tubulin and represented as percentage of control. A representative blot is shown for each protein with arrows corresponding to P2X7 (~75 kDa) and P2X4 (~60 kDa) bands. Luminometric analysis (C) shows ATP concentrations (mol/L) in cell-free supernatants in response to high glucose and palmitate at 0, 24, and 48 h. n = 3 to 6 independent experiments each done in replicates; *p ≤ 0.05.

P2X7 and P2X4 are implicated in mediating immune responses in the central nervous system and in inflammatory cells [16,17,19,20,30–32]. We therefore first investigated the effects of high glucose and palmitate (30 mmol/L glucose and 150 μmol/L palmitate conjugated to fatty acid-free BSA) on the expression of these receptors in endothelial cells. Exposure of HUVECs to high glucose and palmitate increased transcript levels (Fig 1A; 24 h) of *P2X7* (3.3±0.4-fold; *p* = 0.002) and *P2X4* (3.6±0.2-fold; *p* = 0.002) as well as their protein levels (Fig 1B; 48 h), which were increased by 1.46±0.1-fold (*p* = 0.008) and 1.49±0.08-fold (*p* = 0.0006), respectively. The protein bands for P2X4 [29] and P2X7 (~75 kDa; data not shown) were validated with pre-adsorption of the antibodies with the respective control antigens. We next tested if high glucose and palmitate could affect levels of eATP in the HUVECs and indeed, we observed a significant increase in the relative luminescence, which is proportional to the amount of ATP. When compared to vehicle control, the supernatants from cells exposed to high glucose and palmitate showed a 3.6-fold; *p* = 0.005 and 7.7-fold; *p* < 0.0001 increase at 24 h and 48 h, respectively (Fig 1C). However, the elevated levels of ATP were not evident when cells were exposed to mannitol (30 mmol/L) (data not shown).

Since endothelial cells are known to release multiple inflammatory mediators in response to hyperglycemic and dyslipidemic conditions [8,28], we next determined the levels of cytokines, chemokines, and adhesion molecules in the HUVECs under high glucose and palmitate conditions. As shown in S1 Table, we observed a several fold increase in transcript levels of different classes of genes including cytokines (*IL-1β*, *IL-6*), chemokines (*IL-8*), cyclooxygenase (*PTGS2*), inflammasome mediators (*CASP1*, *CASP5*, *TXNIP*), and vascular adhesion molecules (*ICAM-1*, *VCAM-1*). Moreover, the effect of high glucose was not due to hyperosmolarity, as mannitol had no effect on gene expression (S2 Table).

### Blocking P2X7 and P2X4 attenuates high glucose and palmitate-induced cytokines, chemokine, and cyclooxygenase expression

**Fig 2:**
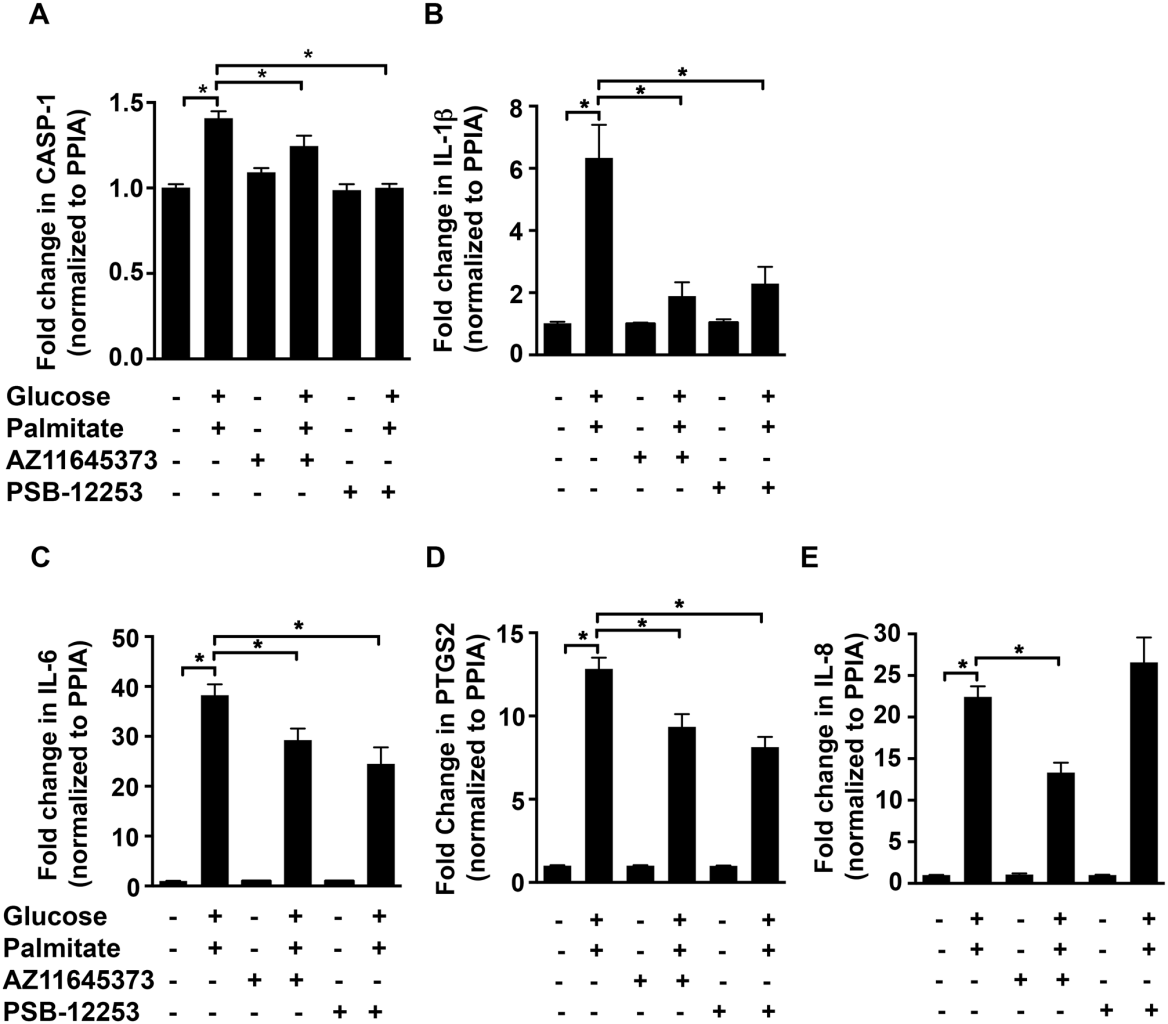
P2X7 and P2X4 antagonists block high glucose and palmitate-induced expression of inflammatory genes. qRT-PCR analysis shows high glucose and palmitate-induced (24 h) transcript levels of *CASP1* (A), *IL-1β* (B), *IL-6* (C), *PTGS2* (D), and *IL-8* (E) in the presence or absence of the P2X7 (AZ11645373) and P2X4 (PSB-12253) antagonists. The transcript levels were normalized to the housekeeping gene, *PPIA*. n = 3 to 4 independent experiments each done in replicates; *p ≤ 0.05.

**Fig 3:**
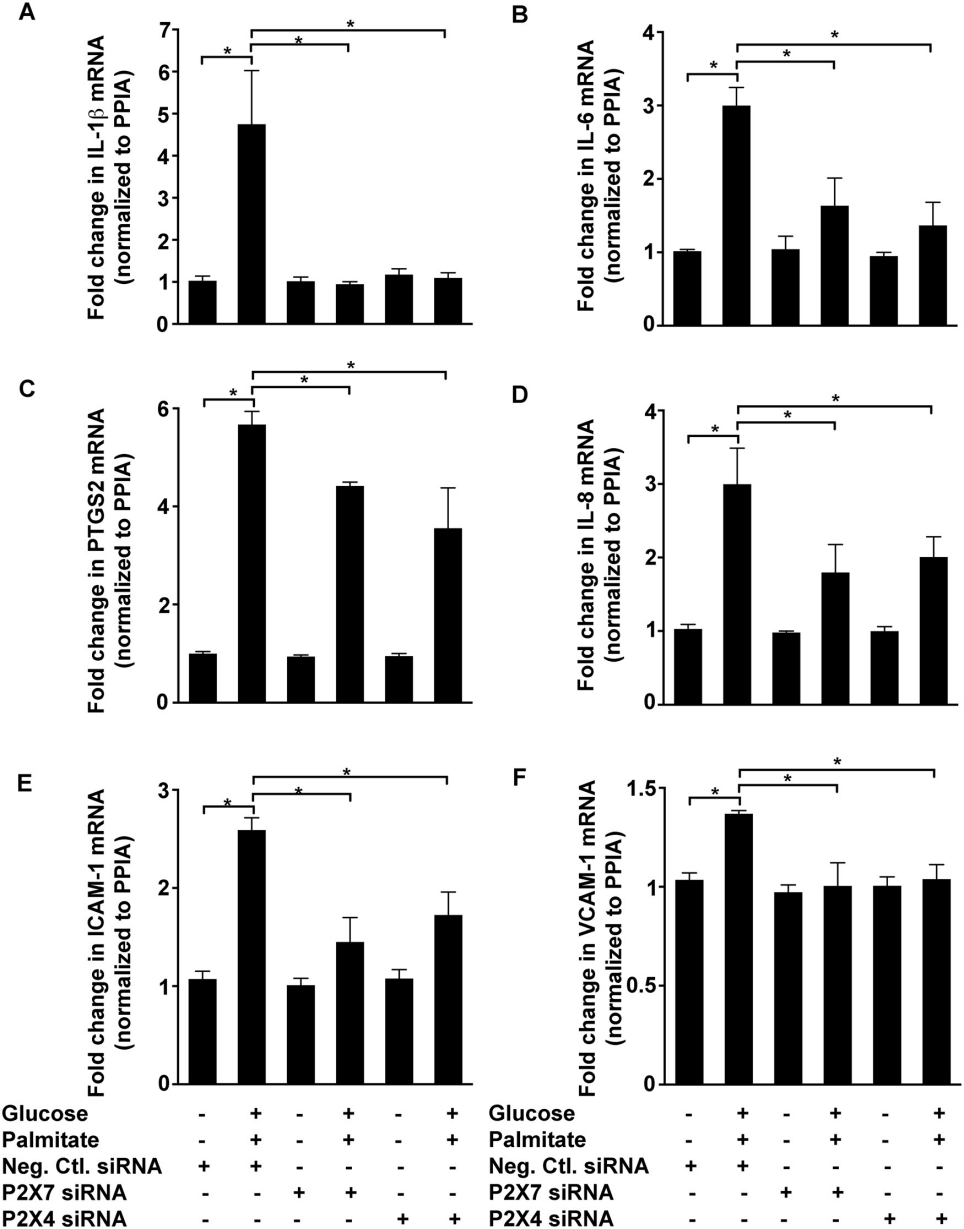
Knockdown of P2X7 and P2X4 inhibit high glucose and palmitate-induced expression of inflammatory genes. siRNA knockdown of P2X7 and P2X4 in the HUVECs inhibits high glucose and palmitate-induced (24 h) gene expression of *IL-1β* (A), *IL-6* (B), *PTGS2* (C), *IL-8* (D), *ICAM-1* (E), and *VCAM-1* (F). Cells transfected with the negative control siRNA (Neg. Ctl. siRNA) was used as controls and *PPIA* was used as the housekeeping gene to normalize all transcript levels. n = 3 independent experiments each done in replicates; *p ≤ 0.05.

Given the observation that conditions of high glucose and palmitate increased the levels of P2X7 and P2X4 in HUVECs, we first used antagonists to test if they were involved in the induction of inflammatory genes. Antagonizing P2X7 with 100 nmol/L AZ11645373 [33] in HUVECs exposed to high glucose and palmitate, inhibited the upregulation (Fig 2) of *CASP1* (21.6±1.8%; *p* < 0.0001; Fig 2A), *IL-1β* (66.7±7.8%; *p* = 0.0002; Fig 2B), *IL-6* (23.5±6%; *p* < 0.0008; Fig 2C), *PTGS2* (27.2±6%; *p* = 0.0002; Fig 2D), and *IL-8* (40.6±5.3%; *p* < 0.0001; Fig 2E). Moreover, PSB-12253 (1 μmol/L), a previously characterized and validated P2X4 antagonist [29,34], had a significant inhibitory effect on *CASP1* (10.5±4%; *p* = 0.001; Fig 2A), *IL-1β* (59.7±10.2%; *p* = 0.0003; Fig 2B), *IL-6* (36±8.7%; *p* < 0.0001; Fig 2C) and *PTGS2* (36.7±4.8%; *p* < 0.0001; Fig 2D) mRNA. Furthermore, we observed similar inhibitory effects using another P2X7 antagonist (50 μmol/L A438079) (S1 Fig). Next, we used siRNA knockdown of P2X7 and P2X4 to determine their specific role in mediating high glucose and palmitate-induced expression of inflammatory markers in HUVECs. Both P2X4 [29] and P2X7 (S2 Fig; 86% decrease in P2X7 mRNA) siRNA were validated in the HUVECs and the effects observed with these siRNA correlated well with that of the antagonists (Fig 3A–3F). We therefore used AZ11645373 and PSB-12253 for the rest of the study.

**Fig 4:**
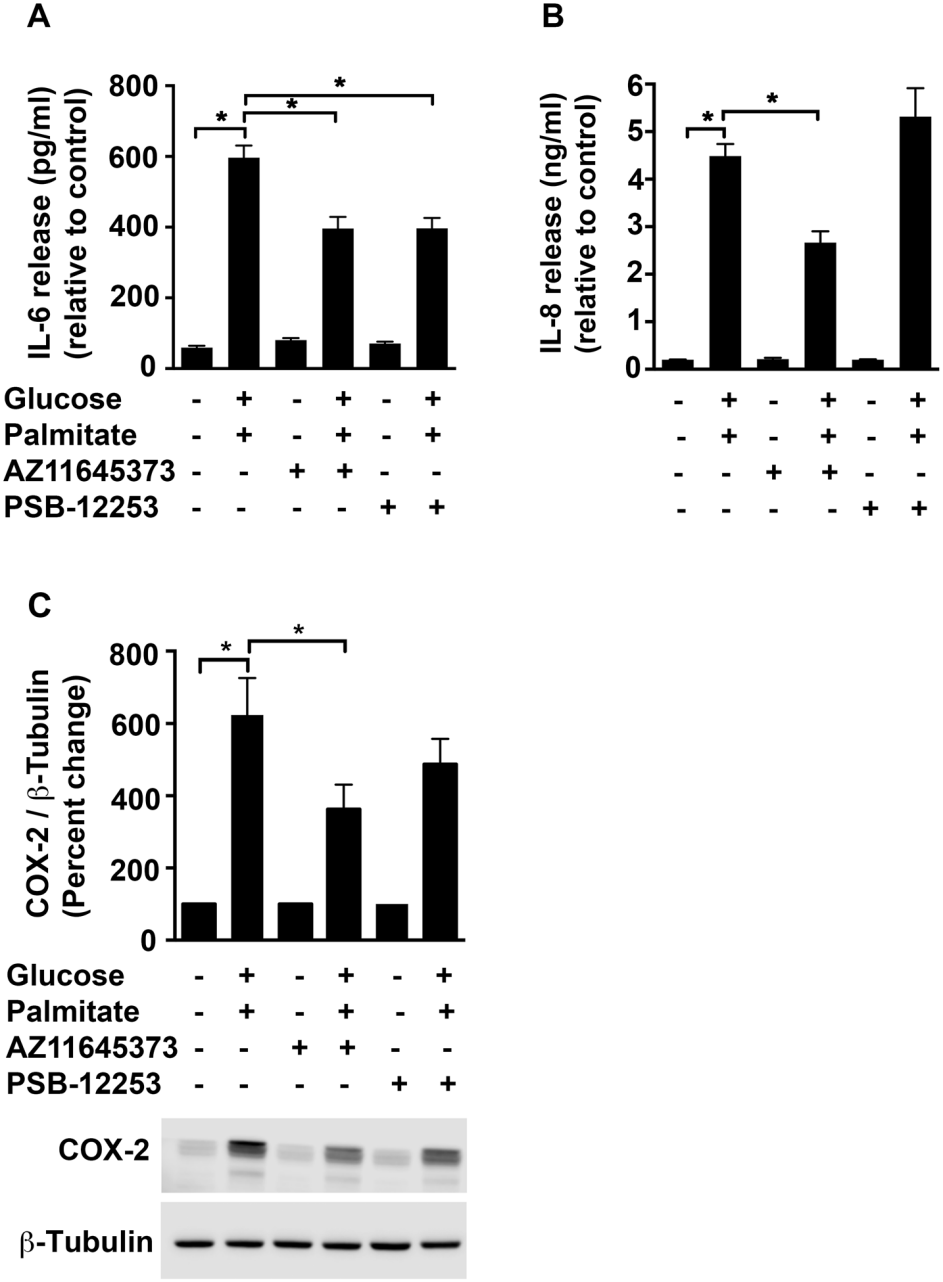
Purinergic modulation of high glucose and palmitate-induced IL-6, IL-8 and COX-2 protein. HUVECs were exposed to high glucose and palmitate (48 h) in the presence or absence of the P2X7 and P2X4 antagonists. The supernatants were analyzed for IL-6 (A) and IL-8 (B) secretion using ELISA. Cell lysates probed for COX-2 (C; 74 kDa) and normalized to β-Tubulin are represented as percentage of control. A representative immunoblot for each protein is depicted. n = 3 to 4 independent experiments each done in replicates; *p ≤ 0.05.

Consistent with the mRNA data, high glucose and palmitate also increased the protein levels of IL-6 in the supernatants by 10-fold (596 pg/ml; *p* < 0.0001; Fig 4A) and IL-8 by 3-fold (4.69 ng/ml; *p* < 0.0001; Fig 4B) when compared to the vehicle control. However, we were unable to detect IL-1β release by ELISA. Although we observed a reduction of IL-6 protein (Fig 4A) with both AZ11645373 (33.6±5.5%; *p* < 0.0001) and PSB-12253 (33.5±5%; *p* < 0.0001) only the former had an inhibitory effect on IL-8 release (18.5±6.2%; *p* = 0.02; Fig 4B) and COX-2 protein levels (41.8±10.9%; *p* = 0.02; Fig 4C).

**Fig 5:**
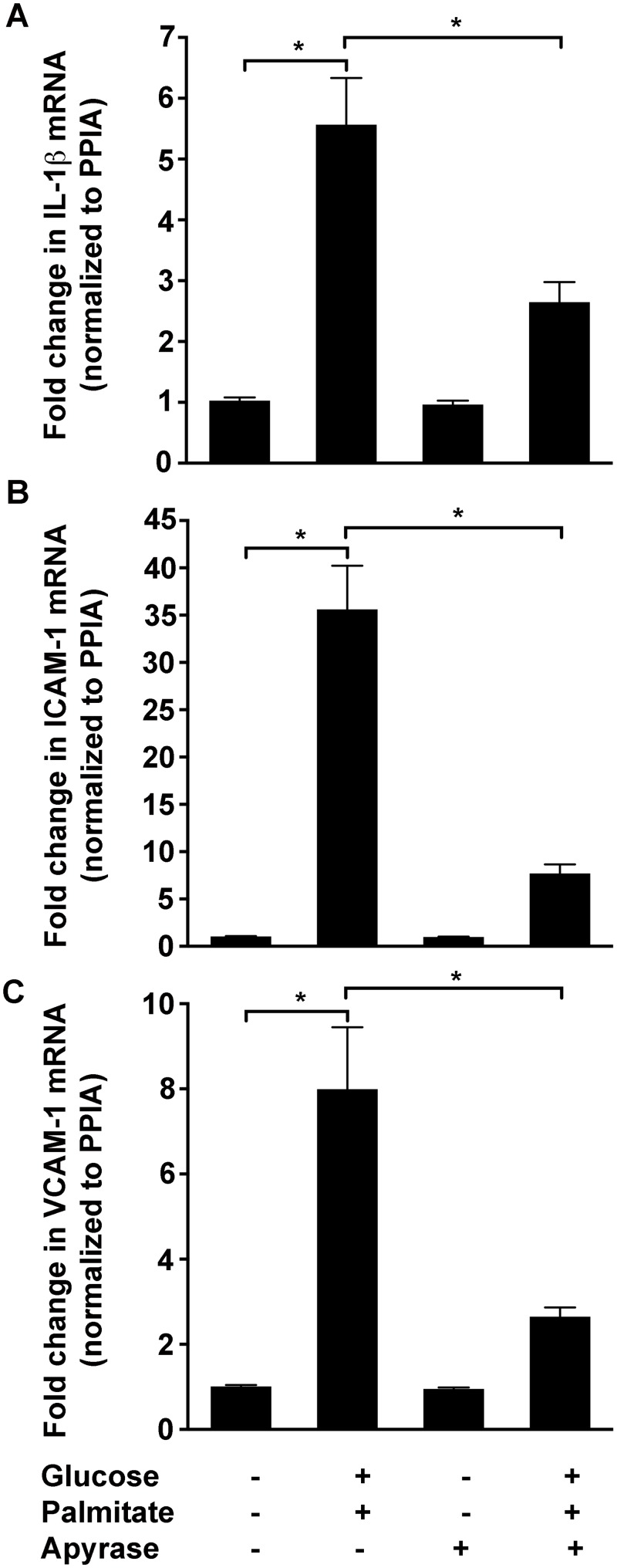
Apyrase reduces high glucose and palmitate-induced expression of inflammatory genes. Apyrase reduces high glucose and palmitate-induced (24 h) expression of *IL-1β* (A), *ICAM-1* (B), and *VCAM-1* (C) in the HUVECs. The transcript levels normalized to *PPIA* are represented as fold change relative to the control. n = 3 independent experiments each done in replicates; *p ≤ 0.05.

To determine if eATP is involved in high glucose and palmitate-induced expression of inflammatory markers, the HUVECs were exposed to apyrase, an enzyme that hydrolyzes ATP to ADP and AMP. While apyrase significantly reduced high glucose and palmitate-induced levels of IL-1β (52.4±5.9%; *p* = 0.004; Fig 5A), ICAM-1 (78.4±2.7%; *p* < 0.0001; Fig 5B), and VCAM-1 (66.9±2.7%; *p* = 0.004; Fig 5C) at 24 h, a decrease in IL-6, IL-8, and PTGS2 was observed only at 48 h (data not shown). Furthermore, exposure of HUVECs to BzATP (300 μM) (S3 Fig) and ATPγS (200 μM) resulted in a significant increase in the transcript levels of IL-1β, which is well in line with prior studies [31,35].

### Effects of high glucose and palmitate on ROS

**Fig 6:**
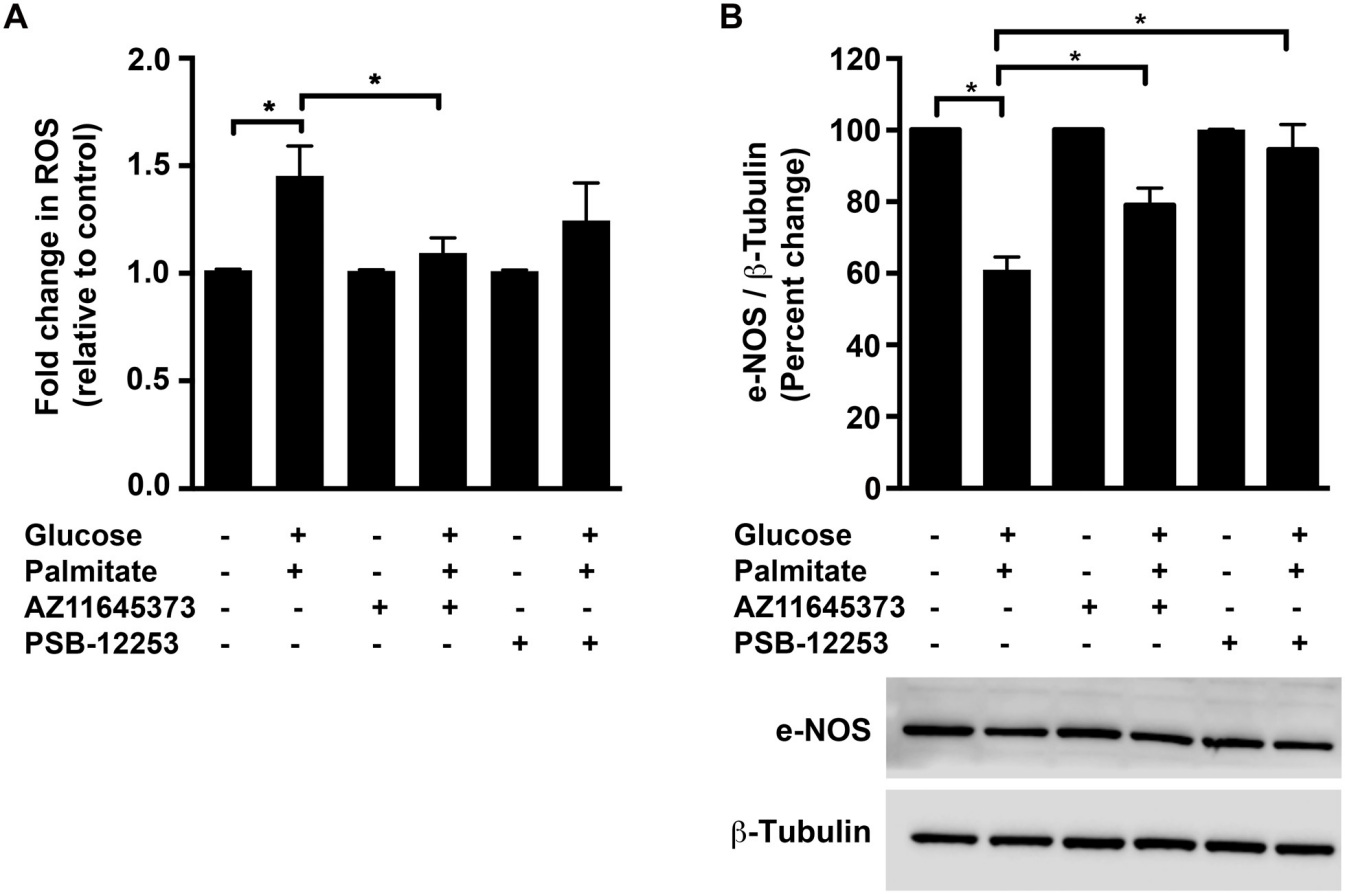
P2X7 mediates high glucose and palmitate-induced ROS and eNOS. Intracellular ROS (A) in the HUVECs exposed to high glucose and palmitate for 24 h was measured with the fluorescent probe, H_2_DCFDA. Immunoblot analysis of eNOS protein (B; 140 kDa) in HUVECs exposed to high glucose and palmitate for 48 h, which was normalized to β-Tubulin and represented as percentage of control. A representative blot for each protein is depicted. n = 5 to 6 independent experiments each done in replicates; *p ≤ 0.05.

Oxidative stress is known to contribute to the development and progression of diabetic vascular complications associated with endothelial dysfunction and activation of the P2X7 has previously been reported to induce ROS in different cell types including epithelial cells, microglia and endothelium-intact aortic rings [15,36,37]. Hence, we investigated the role of P2X7 and P2X4 in high glucose and palmitate-induced oxidative stress using the ROS sensitive dye, H_2_DCFDA. We observed a 1.5±0.1-fold increase (*p* = 0.008; Fig 6A) in ROS levels in response to high glucose and palmitate, which was significantly inhibited by AZ11645373 (24.6±4.8%; *p* = 0.04; Fig 6A) but not by PSB-12253. An important aspect of endothelial dysfunction is reduced nitric oxide (NO) bioavailability, which is due to impaired production of NO by the endothelial cells and/or inactivation of NO by oxidative stress [38,39]. We therefore assessed the effect of high glucose and palmitate on the protein levels of eNOS that is critical for the production of endothelial NO. High glucose and palmitate reduced eNOS levels by about 39% (Fig 6B; *p* < 0.0001), which was reversed by both AZ11645373 (29.6±7.7%; *p* = 0.003; Fig 6B) and PSB-12253 (55±11.4%; *p* < 0.0001; Fig 6B).

### High glucose and palmitate modulates the expression of vascular cell adhesion molecules and endothelial-leukocyte adhesion

Inflammation involves many events that attract leukocytes to injured or infected tissues. The cell adhesion molecules, ICAM-1 and VCAM-1, expressed on endothelial cells are well-established markers of activated endothelial cells that mediate the adhesion of leukocytes to the vascular wall. We therefore tested the hypothesis that P2X7 and P2X4 mediate high glucose and palmitate-induced cell adhesion molecule expression in HUVECs.

**Fig 7:**
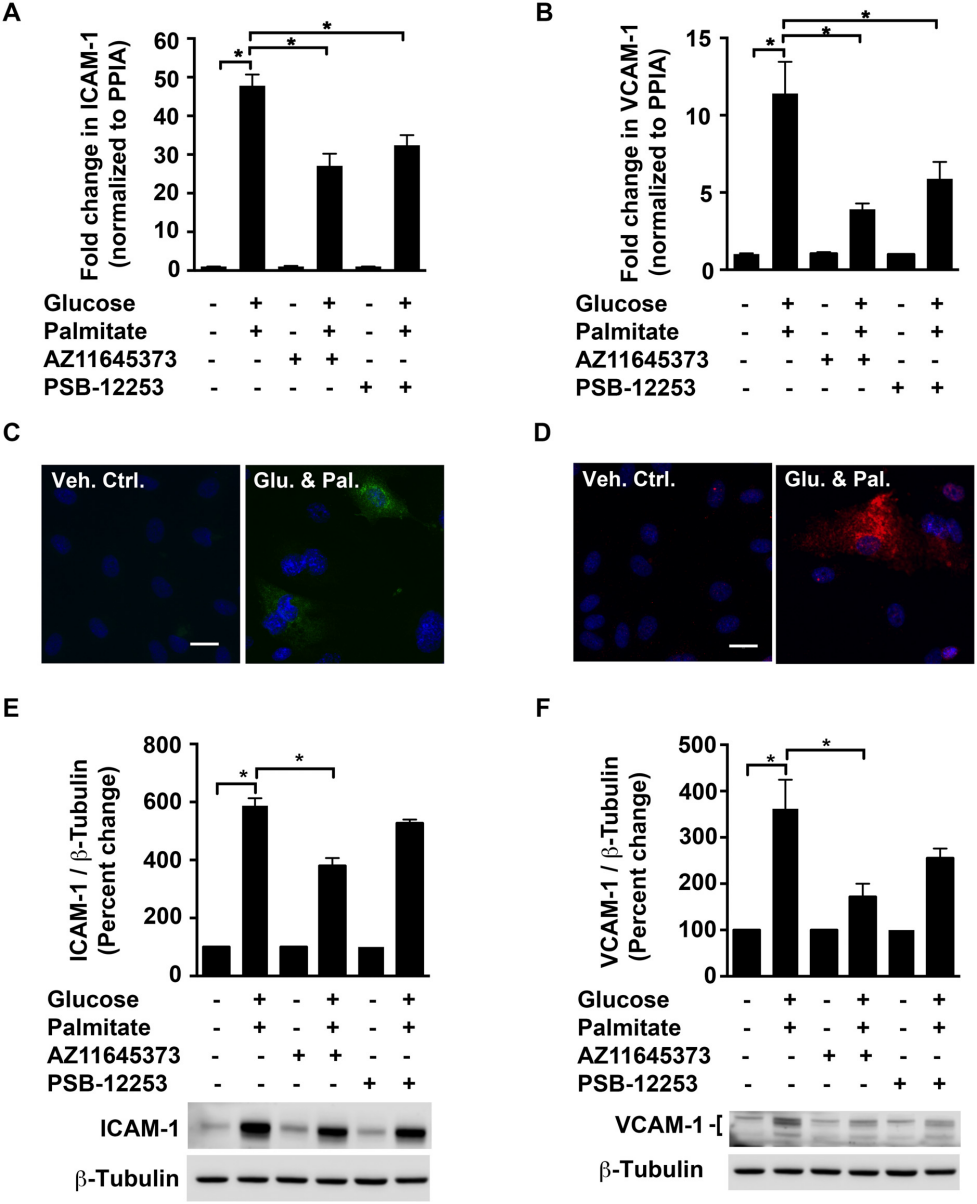
P2X7 mediates high glucose and palmitate-induced increase in ICAM-1 & VCAM-1. qRT-PCR analysis shows increased *ICAM-1* (A) and *VCAM-1* (B) mRNA in response to high glucose and palmitate (24 h). The transcript levels were normalized to the housekeeping gene, *PPIA*. Representative epifluorescent images (x40 objective) of HUVECs exposed to high glucose and palmitate for 48 h show immunostaining for ICAM-1 (C; green) and VCAM-1 (D; red). The nuclei (blue) were visualized with NucBlue ready probe. Immunoblot analysis shows ICAM-1 (E; ~90 kDa)) and VCAM-1 (F) proteins normalized to β-Tubulin and are expressed as percentage of control with representative blots for each protein depicted. For VCAM-1, the antibody recognized multiple bands (a doublet in samples exposed to high glucose and palmitate and an additional band between 95–120 kDa), which were quantified and represented in the blot. n = 4 to 5 independent experiments each done in replicates; *p ≤ 0.05. Scale bar—20 μM.

We observed an increase in both mRNA and protein expression of ICAM-1 (47.8±2.9-fold; *p* < 0.0001; Fig 7A and 7C) and VCAM-1 (11.4±2-fold; *p* < 0.0001; Fig 7B and 7D) in HUVECs subjected to high glucose and palmitate when compared to vehicle control. AZ11645373 significantly reduced the high glucose and palmitate-induced increase of mRNA and protein of ICAM-1 (49.2±4.3%; *p* < 0.0001; Fig 7A and 35.3±4.6%; *p* < 0.0001; Fig 7E) as well as VCAM-1 (65.7±3.3%; p = 0.0002; Fig 7B and 52.6±8%; *p* = 0.003; Fig 7F). While PSB-12253 had a significant inhibitory effect at the transcript level of ICAM-1 (25.7±5.7%; *p* = 0.003; Fig 7A), and of VCAM-1 (43.2±9.9%; *p* = 0.04; Fig 7B), we observed a trend towards a decrease in the levels of these proteins (10.3±2.1%; *p* = 0.08; Fig 7E and 29.4±5.7%; *p* = 0.14; Fig 7F).

**Fig 8:**
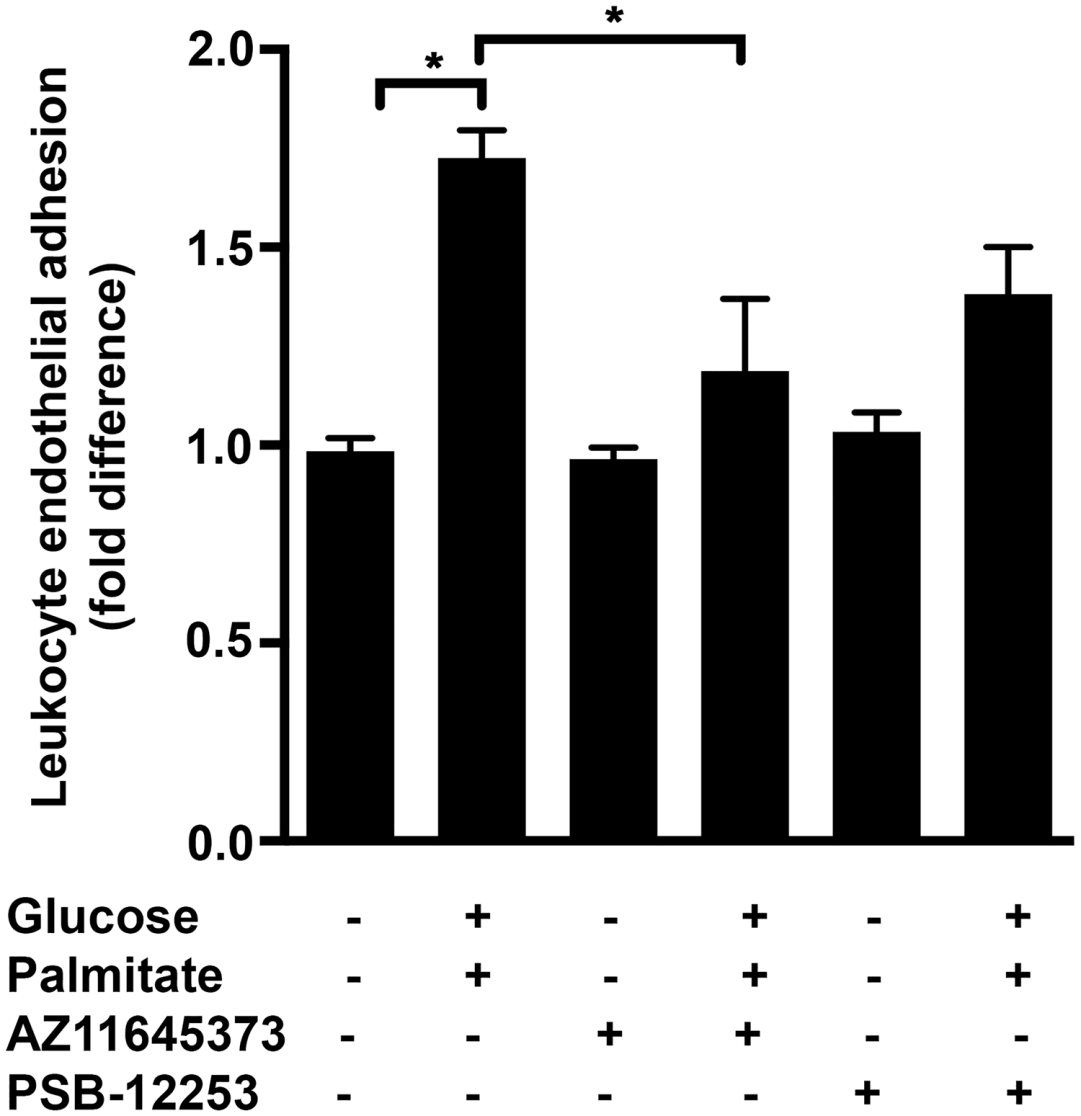
P2X7 mediates high glucose and palmitate-induced increase in leukocyte adhesion. HUVEC monolayers seeded in 96-well plates were exposed to high glucose and palmitate in the presence or absence of receptor antagonists for 48 h. Leukocytes labeled with LeukoTracker were allowed to attach for 90 mins after which adherent cells were lysed and the fluorescence was measured at an excitation and emission wavelengths of 480 nm and 520 nm, respectively. n = 4 independent experiments each done in replicates; *p ≤ 0.05.

Exposure of HUVECs to 48 h high glucose and palmitate significantly increased the leukocyte adherence by more than 1.7±0.07-fold (Fig 8; *p* = 0.0001), which was significantly attenuated by AZ11645373 (40.2±1.7%; *p* = 0.003; Fig 8). On the other hand exposure of the cells to PSB-12253 only resulted in a trend towards a decrease (20±7%; *p* = 0.07; Fig 8). This demonstrates high glucose and palmitate-induced endothelial dysfunction characterized by the increased adhesiveness in a mainly P2X7-dependent manner.

### High glucose and palmitate increases endothelial cell permeability in a P2X7- and P2X4-dependent manner

**Fig 9:**
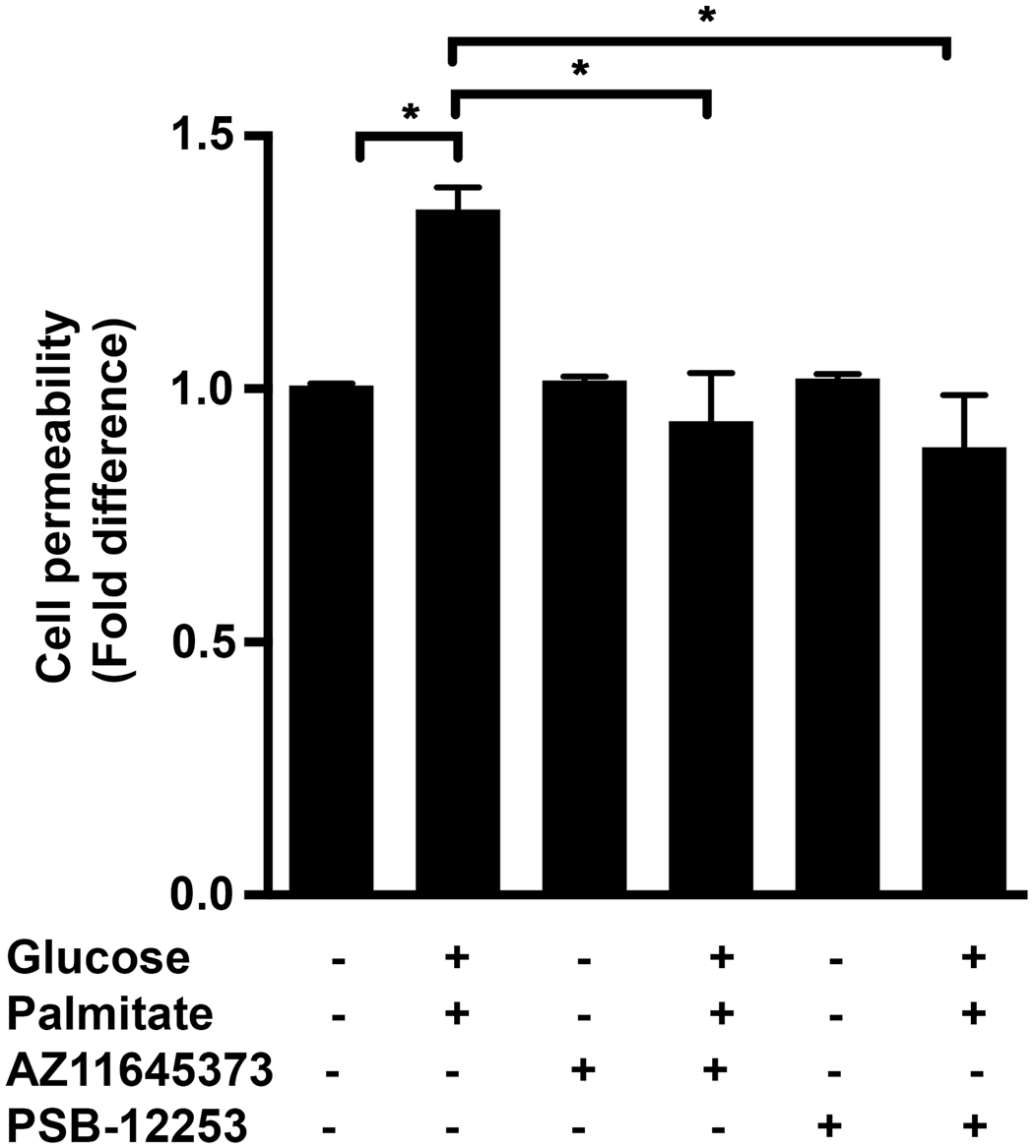
P2X7 and P2X4 mediate high glucose and palmitate-induced cell permeability. HUVEC monolayers were seeded in 24-well transwell inserts (0.4 μm) and exposed to high glucose and palmitate in the presence or absence of receptor antagonists for 48 h. 1 mg/ml FITC-dextran (MW 40,000 Da) was added in the upper well and the media collected from the lower well after 1 h. FITC-dextran flux was assessed by measuring the fluorescence at an excitation and emission wavelengths of 485 nm and 530 nm, respectively. Percent permeability was calculated and represented as fold difference relative to controls. n = 5 experiments each done in replicates; *p ≤ 0.05.

The integrity of endothelial cell junctions and the maintenance of barrier function are important for vascular homeostasis. Disruption of endothelial cell permeability is associated with several systemic diseases including diabetes, and cardiovascular disease (CVD) [40,41]. We tested the hypothesis that high glucose and palmitate increases cell permeability in a P2X7- and P2X4-dependent manner using the FITC-dextran assay in the HUVEC monolayer cultured on the transwell membrane inserts. We observed a 1.4±0.04-fold increase (*p* = 0.001) in the permeability as assessed by the increased diffusion of FITC-dextran when the HUVECs were exposed to high glucose and palmitate (Fig 9). This effect was inhibited by AZ11645373 (31±7%; *p* = 0.0002; Fig 9) and PSB-12253 (34.6±7.5%; *p* < 0.0001; Fig 9) indicating the importance of these receptors in maintaining the barrier integrity.

### P2X7-dependent effects of high glucose and palmitate is mediated by p38-MAPK

**Fig 10:**
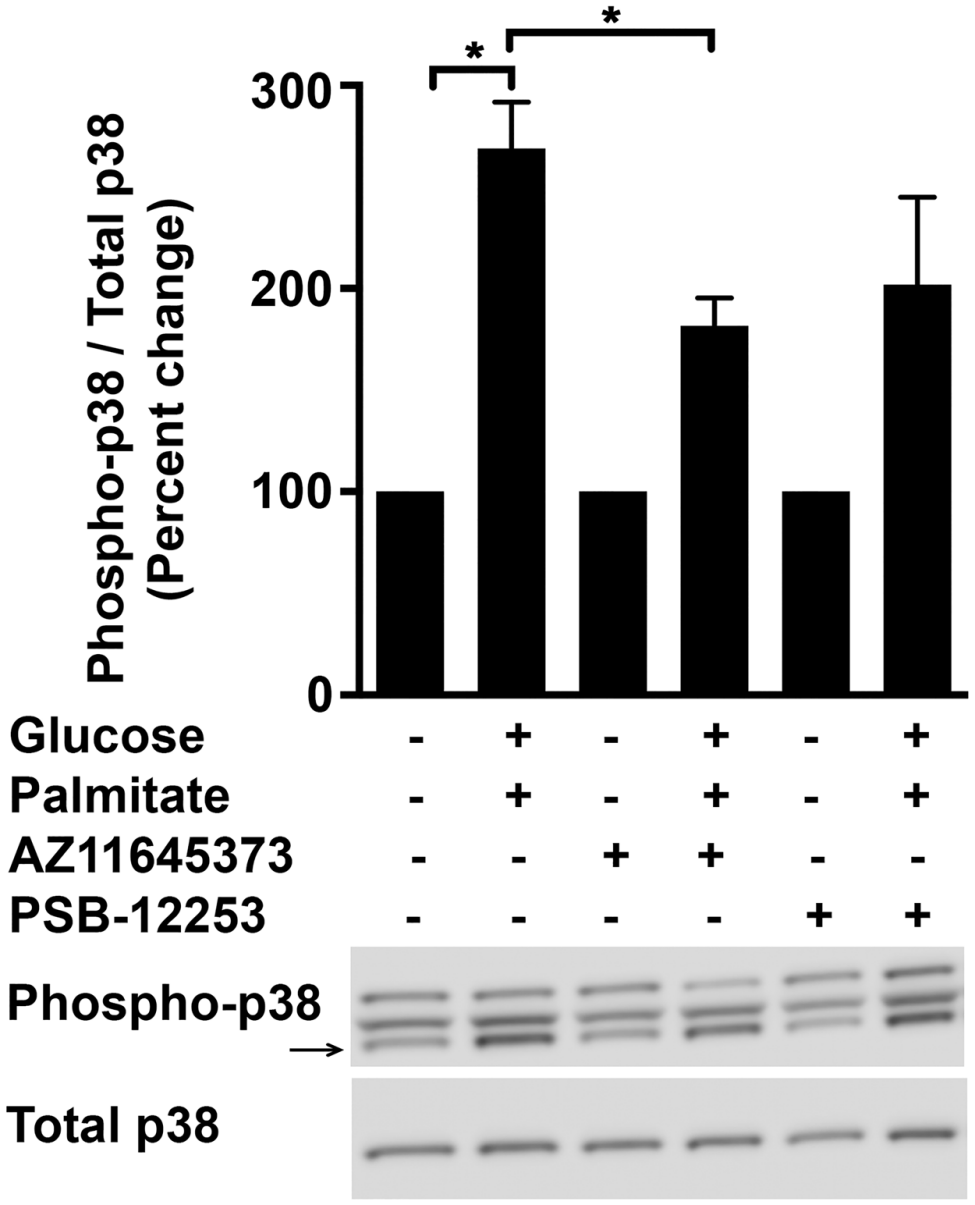
High glucose and palmitate induces activation of p38-MAPK in a P2X7-dependent manner. HUVECs were exposed to high glucose and palmitate for 48 h and immunoblot analysis of cell lysates shows increased phosphorylated-p38-MAPK that was normalized to total p38-MAPK and expressed as percentage of control. A representative immunoblot for each protein is shown. n = 3 experiments each done in replicates; *p ≤ 0.05.

It is well documented that various stimuli including metabolic stressors and ROS activate p38-MAPK, which is implicated in modulating inflammatory responses in different cell types including endothelial cells [42–47]. Indeed p38-MAPK (Fig 10; 268.9±23%; *p* = 0.0003) was activated in HUVECs subjected to high glucose and palmitate. As positive control for the activation of p38-MAPK, we exposed the HUVECs to 250 ng/ml of lipopolysaccharide (LPS) (data not shown). The activation of p38-MAPK was inhibited by AZ11645373 (32.5±5%; *p* = 0.03) but not by PSB-12253, thus implicating P2X7 in this signaling cascade.

## Discussion

**Fig 11:**
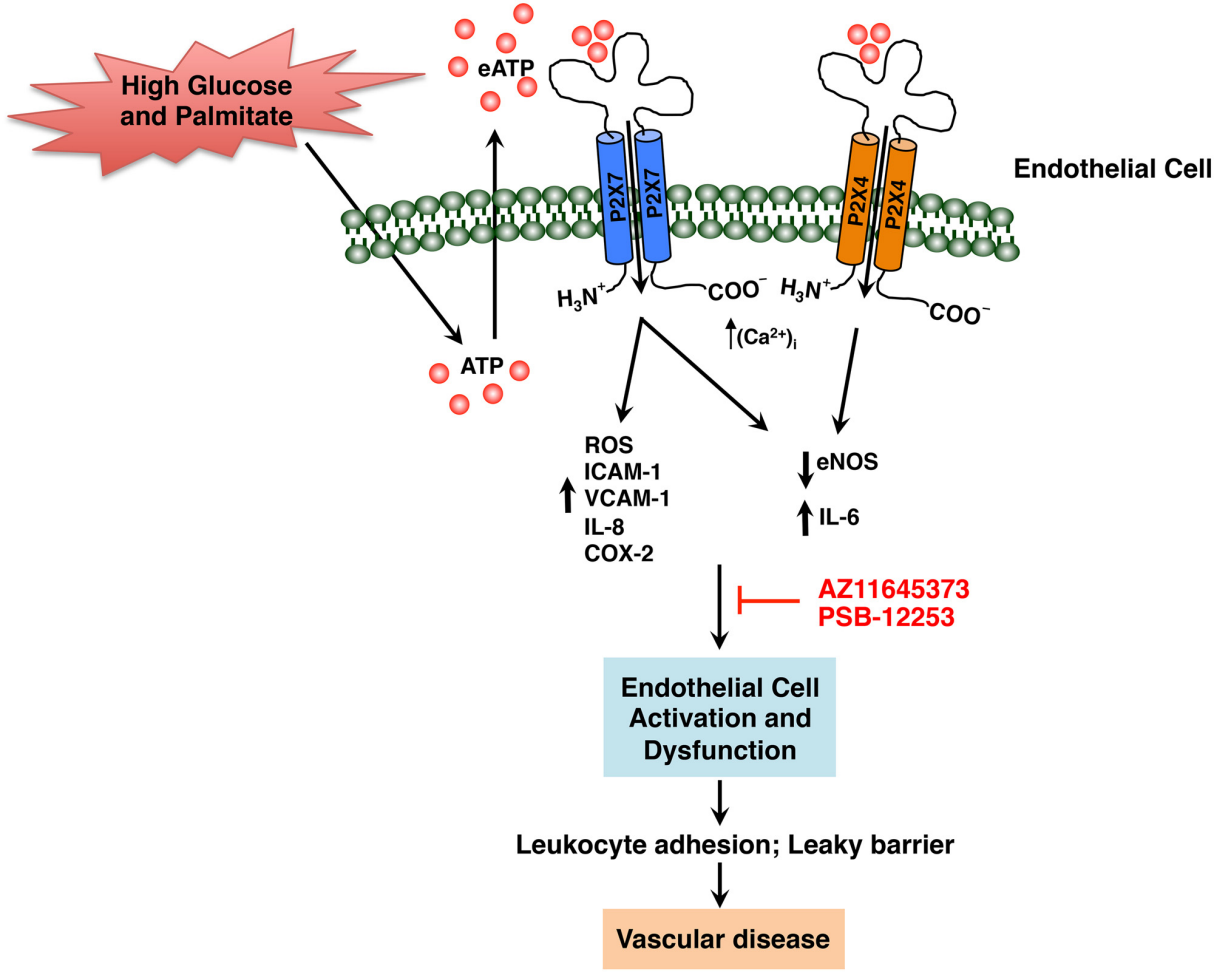
Schematic representation of high glucose and palmitate-induced endothelial cell activation and dysfunction modulated in part by P2X7 and P2X4. Exposure of HUVECs to high glucose and palmitate results in the activation of the P2X7 and P2X4 causing (i) increased intracellular ROS and reduced eNOS contributing to endothelial cell dysfunction, and (ii) increased expression of IL-6, ICAM-1, VCAM-1, IL-8, and COX-2 resulting in endothelial cell activation. Blocking the P2X7 and P2X4 with AZ11645373 and PSB-12253, respectively, had a partial inhibitory effect on ROS as well as the inflammatory molecules with decreased leukocyte adhesion and vascular permeability. This is suggestive of the possible roles for P2X7 and P2X4 in modulating high glucose and palmitate-induced endothelial cell dysfunction, an early determinant of vascular disease.

Endothelial dysfunction is an early determinant of vascular disease progression and is common to the pathogenesis of diabetes and CVD. Dietary factors contribute to the etiology and pathophysiology of the dysfunctional endothelium characterized by endothelial cell activation, augmented pro-inflammatory events, and attenuated barrier function increasing the risk of atherosclerosis. High glucose and palmitate is a physiological challenge that alters endothelial gene expression and function. In the present study, we hypothesized that high glucose and palmitate contribute to endothelial cell dysfunction through exocrine/paracrine activation of P2X7 and P2X4. Our results demonstrate high glucose and palmitate-mediated up-regulation of P2X7 and P2X4 and an increase in pro-inflammatory genes *CASP1*, *IL-1β*, *1L-6*, *IL-8*, *PTGS2*, *ICAM-1*, and *VCAM-1*. While intracellular ROS and the protein levels of IL-6, IL-8, COX-2, ICAM-1, and VCAM-1 were increased in a P2X7-specific manner, high glucose and palmitate-mediated decrease in eNOS protein was partly dependent on both P2X7 and P2X4. Concurrent with the increased expression of the adhesion molecules and IL-8, we observed enhanced leukocyte-endothelial cell adhesion that was P2X7-dependent. On the other hand, the high glucose and palmitate-induced endothelial cell permeability turned out to be dependent on P2X7 as well as P2X4. Finally, we also show that high glucose and palmitate activates p38-MAPK in a P2X7-dependent manner. Based on these findings, a model that illustrates the role of P2X7 and P2X4 in modulating the effects of high glucose and palmitate on endothelial cell function is provided in Fig 11.

The extracellular nucleotides acting via P2 receptors modulate intracellular signaling cascades in several pathophysiological processes including inflammation and metabolism [48–51]. In view of this, we observed high glucose and palmitate to increase eATP levels in the HUVECs. The inhibitory effect of apyrase on the induction of proinflammatory genes indicates that these responses are, at least in part, dependent on an autocrine/paracrine stimulation of P2X7 and P2X4. However, the eATP concentrations measured in the supernatants were all below the concentration range necessary for receptor activation. The most likely explanation for this discrepancy is that the release and receptor activation takes place simultaneously at the plasma membrane level before dilution and ectonucleotidase-mediated degradation reduces the concentration of eATP. It is also tempting to speculate that the high glucose and palmitate could directly affect the sensitivity of these receptors to ATP. This idea is supported by reports that show either changing the membrane fluidity by cholesterol removal or by addition of lipids increases the potency of ATP at the P2X7 [52,53]. The exposure of HUVECs to BzATP and ATPγS alone did not completely mimic the effects of high glucose and palmitate and it can therefore be concluded that ATP plays a modulatory role by enhancing an ongoing proinflammatory response. Furthermore, our observation that high glucose and palmitate resulted in upregulation of P2X7 and P2X4 expression in the HUVECs can be explained by indirect effects, for instance, via the proinflammatory cytokines as previously reported [35,54–56].

P2X7 is reported to play a role in diabetes-related pathologies of the fibroblasts, retina, and renal inflammation [18,23,57] while P2X4 is implicated in mediating inflammatory responses during neuropathic pain [32,58,59]. Interestingly, both glucose and free fatty acids activate P2X7 in human islets thereby regulating β cell mass and function [22]. Considering the central role of these receptors in inflammatory pathologies our novel observations of high glucose and palmitate induced effects in endothelial cells modulated in part by P2X7 and P2X4 is therefore important for understanding their role in vascular disease.

It is well documented that in subjects with metabolic syndrome, elevated plasma levels of free fatty acids contribute to insulin resistance with a resulting increase in glucose levels and chronic sub-acute inflammation [60,61]. Inflammatory responses coupled to P2 receptor stimulation have previously been reported to result in endothelial cell activation [35,45]. Consistent with these reports we show high glucose and palmitate to increase the transcript levels of *IL-1β*, *IL-6*, and *IL-8* in HUVECs. Although we observed P2X7 to mediate high glucose and palmitate-induced transcript levels of *CASP1* and *IL-1β* (S1 Table), we did not see differences in caspase-1 protein and IL-1β was undetectable in the supernatants. We could detect IL-1β in supernatants of cells exposed to LPS but were unable to detect IL-1β in cells exposed to glucose and palmitate even with a high sensitive ELISA kit. This is possibly due to the levels being below the threshold for detection and is in line with the study by Staiger *et al*., who show that neither palmitate nor stearate is able to induce IL-1β secretion in human coronary artery endothelial cells (HCAECs) [62]. Furthermore, combinations of potent inflammatory stimuli such as LPS, IFNγ, TNFα, IL-1β, and P2X agonists (3 mM ATP and 300 μM BzATP) resulted in only low picogram quantities of secreted IL-1β in HUVECs suggestive of a low synthetic capacity compared to immune cells [35]. Moreover, a speculation is that the elevated pro-inflammatory state, caused by high glucose and palmitate, may reflect the heightened predisposition of diabetic patients to develop an inflammatory response by bacterial or viral infections [63].

The observation that HUVECs, exposed to high glucose and palmitate, display an increased release of IL-6 is well in line with previous studies that have associated elevated IL-6 in response to palmitate in HCAECs as well as high circulating levels in patients with diabetes and CVD [28,64–66]. Interestingly, we show significantly reduced IL-6 levels by antagonizing and knocking down the P2X7 and P2X4. IL-6 is a pleiotropic cytokine that has a role not only in the acute phase response but also in chronic inflammation and endothelial dysfunction. Downstream effects of IL-6 in a pro-inflammatory context include increased expression of adhesion molecules, plasminogen activator inhibitor-1, and release of chemokines, which are all known risk factors for hypertension, CVD, and diabetes. In fact, in our *in vitro* model, we observe P2X7-specific increase in the levels of IL-8, ICAM-1, and VCAM-1, which is supported by reports that P2X7 knock out mice display altered cytokine production and attenuation of leukocyte-mediated inflammatory responses [67,68]. Moreover, recent studies that investigated the effects of single nucleotide polymorphisms (SNPs) in the human P2X7 suggest a role for this receptor in cytokine release from immune cells [69,70]. Furthermore, SNPs in P2X7 are shown to be associated with decreased risk of ischemic stroke as well as ischemic heart disease in smokers [71]. Given that we observe differential but significant inhibitory effects with the P2X4 antagonist and siRNA, we cannot exclude its role in addition to P2X7 in modulating high glucose and palmitate-induced inflammatory responses thus supporting our hypothesis that both the receptors play a role in endothelial cell activation and dysfunction.

Exposure of endothelial cells to LPS, cytokines, and chemokines can result in elevated oxidative stress [72,73]. Indeed, we observe a P2X7-dependent increase in ROS that may add to the high glucose and palmitate-induced inflammation in HUVECs. Our hypothesis is then that hyperglycemia and excess free fatty acids can induce a vicious cycle of events in the vascular wall involving low-grade inflammation, oxidative stress, pro-coagulant state, and impaired vasodilation all of which constitute endothelial dysfunction in the early stages of vascular disease. Endogenous endothelial NO is known to inhibit endothelial cell activation [74]. In this context we observed a decrease in eNOS in the HUVECs exposed to high glucose and palmitate, which in part was rescued by both P2X7 and P2X4 antagonists. COX-2 an enzyme known to modulate superoxide levels is reported to be elevated in response to palmitate as well as to high glucose [75]. Indeed, we report an increase in COX-2 levels in response to high glucose and palmitate that was inhibited by blocking both P2X7 and P2X4 suggesting their involvement in this process.

The endothelial cells of the vascular wall have an indispensable role in leukocyte recruitment and the maintenance of barrier function by producing inflammatory mediators as well as expressing adhesion molecules. While VCAM-1 is involved in the recruitment of leukocytes, ICAM-1 and IL-8 are important in the transmigration of these cells through the vessel wall [8,76,77]. Our findings that high glucose and palmitate-induced expression of VCAM-1, ICAM-1, and IL-8 correlates well with enhanced leukocyte adhesion, which was found to be primarily dependent on P2X7 thus highlighting the importance of this receptor in guarding the vessel wall from leukocyte extravasation. An interesting additional result was that enhanced cell permeability induced by high glucose and palmitate was mediated not only by P2X7 but also by P2X4. Glass *et al*., have previously reported that P2X4 associate with VE-cadherin at endothelial adherens junctions [78]. This is interesting as VE-cadherin is integral in maintaining endothelial barrier stability and in regulating cellular junctions. Furthermore, the possibility of a physical interaction does exist as P2X7 have been described to interact with P2X4 in macrophages [15,16]. In light of these reports, our findings suggest that the high glucose and palmitate-mediated activation of the P2X7 and P2X4 play a role in endothelial barrier function.

p38-MAPK is an extracellular-regulated kinase that has an important role in mediating inflammatory and stress responses in endothelial cells. Both high glucose and palmitate are known activators of this signaling pathway with functional consequences in mediating inflammation, leukocyte recruitment, and subsequent endothelial dysfunction [42,43,45]. In addition prior reports show P2X7-mediated IL-6 release and early brain injury after subarachnoid hemorrhage to be dependent on the activation of p38-MAPK [20,79]. Our data demonstrate the activation of this well studied stress kinase by high glucose and palmitate to be dependent on P2X7 in HUVECs. Thus, this study provides new insights into a possible purinergic mechanism in response to high glucose and palmitate in endothelial dysfunction.

In conclusion, this study highlights the importance of P2X7 and P2X4 in part mediating metabolic stress-induced endothelial dysfunction, an essentially unexplored area. The novel observations that these receptors is involved in high glucose and palmitate-mediated endothelial dysfunction implicates these ligand-gated ion channels in the pathogenesis of vascular disease and as potential therapeutic targets. Downstream signaling of these P2 purinoceptors in the context of endothelial dysfunction may offer further opportunity for the identification of specific targets for intervention and the development of effective therapeutics.

## Supporting Information

S1 Fig P2X7 antagonist (A438079) blocks high glucose and palmitate-induced expression of inflammatory genes. qRT-PCR analysis shows high glucose and palmitate-induced (24 h) transcript levels of *CASP1* (A), *IL-1β* (B), *IL-6* (C), *IL-8* (D), *PTGS2* (E), *ICAM-1* (F) and *VCAM-1* (G) in the presence or absence of A438079. Transcripts were normalized to the housekeeping gene, *PPIA*. n = 3 independent experiments each done in replicates; *p ≤ 0.05.
(TIF)

S2 Fig Validation of the P2X7-specific siRNA. qRT-PCR analysis shows P2X7 mRNA (24 h; normalized to PPIA) to be knocked-down by P2X7-specific siRNA when compared to cells transfected with negative control siRNA (Neg. Ctl. siRNA). n = 3 independent experiments each done in replicates; *p ≤ 0.05.
(TIF)

S3 Fig P2X7 agonists induce expression of IL-1β. BzATP and ATPγS, the P2X7 agonists increase transcript levels (24 h) of *IL-1β*. Transcripts were normalized to the housekeeping gene, *PPIA*. n = 3 independent experiments each done in replicates; *p ≤ 0.05.
(TIF)

S1 Table High glucose and palmitate increases transcript levels of different classes of genes. qRT-PCR analysis show fold change (relative to vehicle control) in high glucose and palmitate-induced (24 h) gene expression of P2 receptors, cytokines, chemokines, cyclooxygenase, mediators of inflammasome, and adhesion molecules normalized to the housekeeping gene (*PPIA*). n = 5 to 9 independent experiments each in replicates; *p* ≤ 0.05.
(DOCX)

S2 Table Mannitol had no effect on the transcript levels of different genes. qRT-PCR analysis show fold change in response to 30 mmol/L mannitol (24 h; relative to vehicle control) in gene expression normalized to housekeeping gene (*PPIA*). n = 3 independent experiments each done in replicates.
(DOCX)
