# Correction: The ATP Receptors P2X7 and P2X4 Modulate High Glucose and Palmitate-Induced Inflammatory Responses in Endothelial Cells

**DOI:** 10.1371/journal.pone.0133346

**Published:** 2015-07-17

**Authors:** Ramasri Sathanoori, Karl Swärd, Björn Olde, David Erlinge

There is an error in panel B of Fig 4. Please view Fig 4 here.

**Fig 4:**
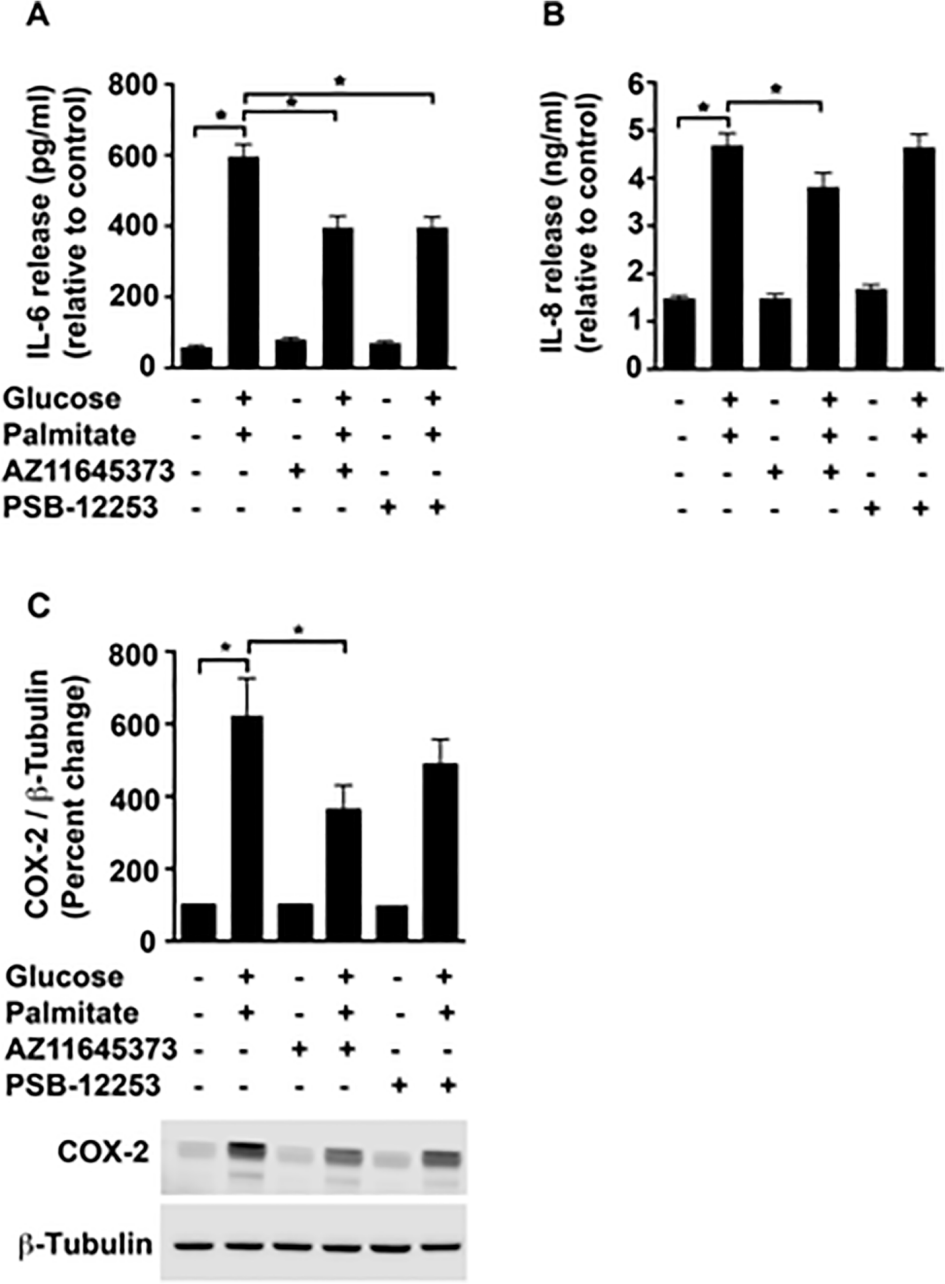
Purinergic modulation of high glucose and palmitate-induced IL-6, IL-8 and COX-2 protein. HUVECs were exposed to high glucose and palmitate (48 h) in the presence or absence of the P2X7 and P2X4 antagonists. The supernatants were analyzed for IL-6 (A) and IL-8 (B) secretion using ELISA. Cell lysates probed for COX-2 (C; 74 kDa) and normalized to β-Tubulin are represented as percentage of control. A representative immunoblot for each protein is depicted. n = 3 to 4 independent experiments each done in replicates; *p ≤ 0.05.
