# Supplementary figures and images for: The ATP Receptors P2X7 and P2X4 Modulate High Glucose and Palmitate-Induced Inflammatory Responses in Endothelial Cells
Source: PLoS One. 2015 May 4;10(5):e0125111. doi: 10.1371/journal.pone.0125111 (PMC4418812; doi:10.1371/journal.pone.0125111)

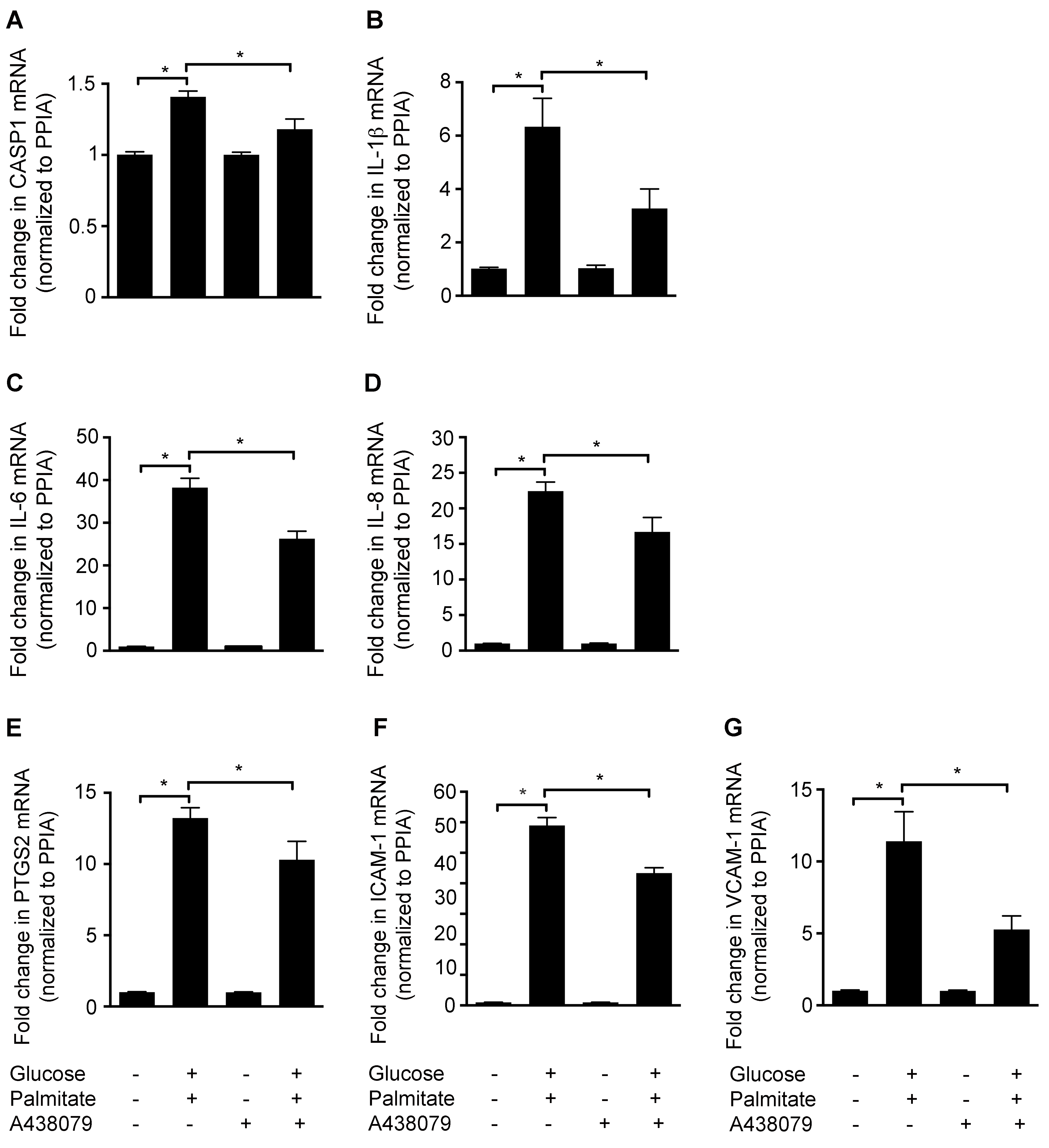

Supplement: S1 Fig — qRT-PCR analysis shows high glucose and palmitate-induced (24 h) transcript levels of CASP1 (A), IL-1β (B), IL-6 (C), IL-8 (D), PTGS2 (E), ICAM-1 (F) and VCAM-1 (G) in the presence or absence of A438079. Transcripts were normalized to the housekeeping gene, PPIA. n = 3 independent experiments each done in replicates; *p ≤ 0.05. (TIF) [file pone.0125111.s001.tif]

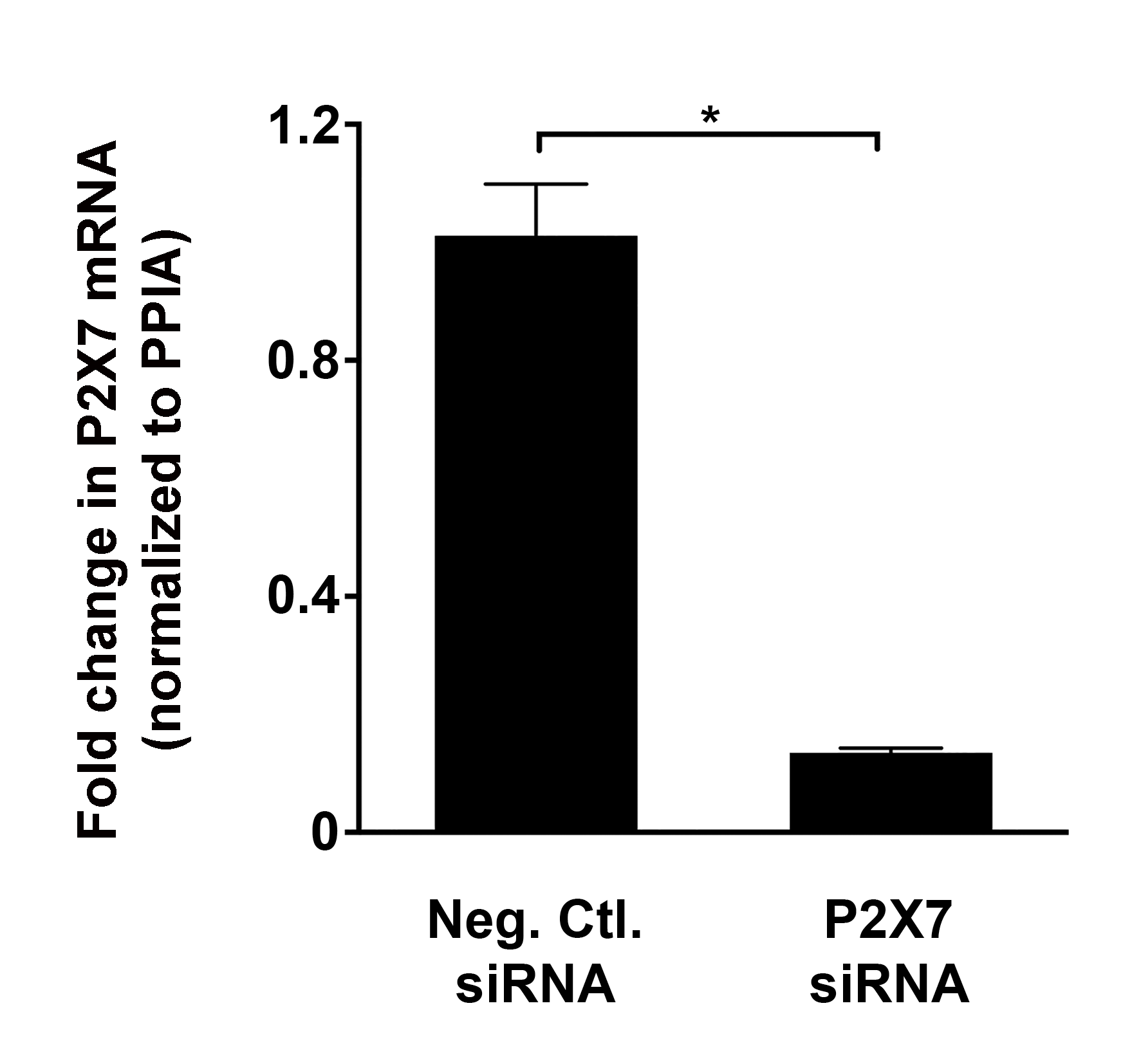

Supplement: S2 Fig — qRT-PCR analysis shows P2X7 mRNA (24 h; normalized to PPIA) to be knocked-down by P2X7-specific siRNA when compared to cells transfected with negative control siRNA (Neg. Ctl. siRNA). n = 3 independent experiments each done in replicates; *p ≤ 0.05. (TIF) [file pone.0125111.s002.tif]

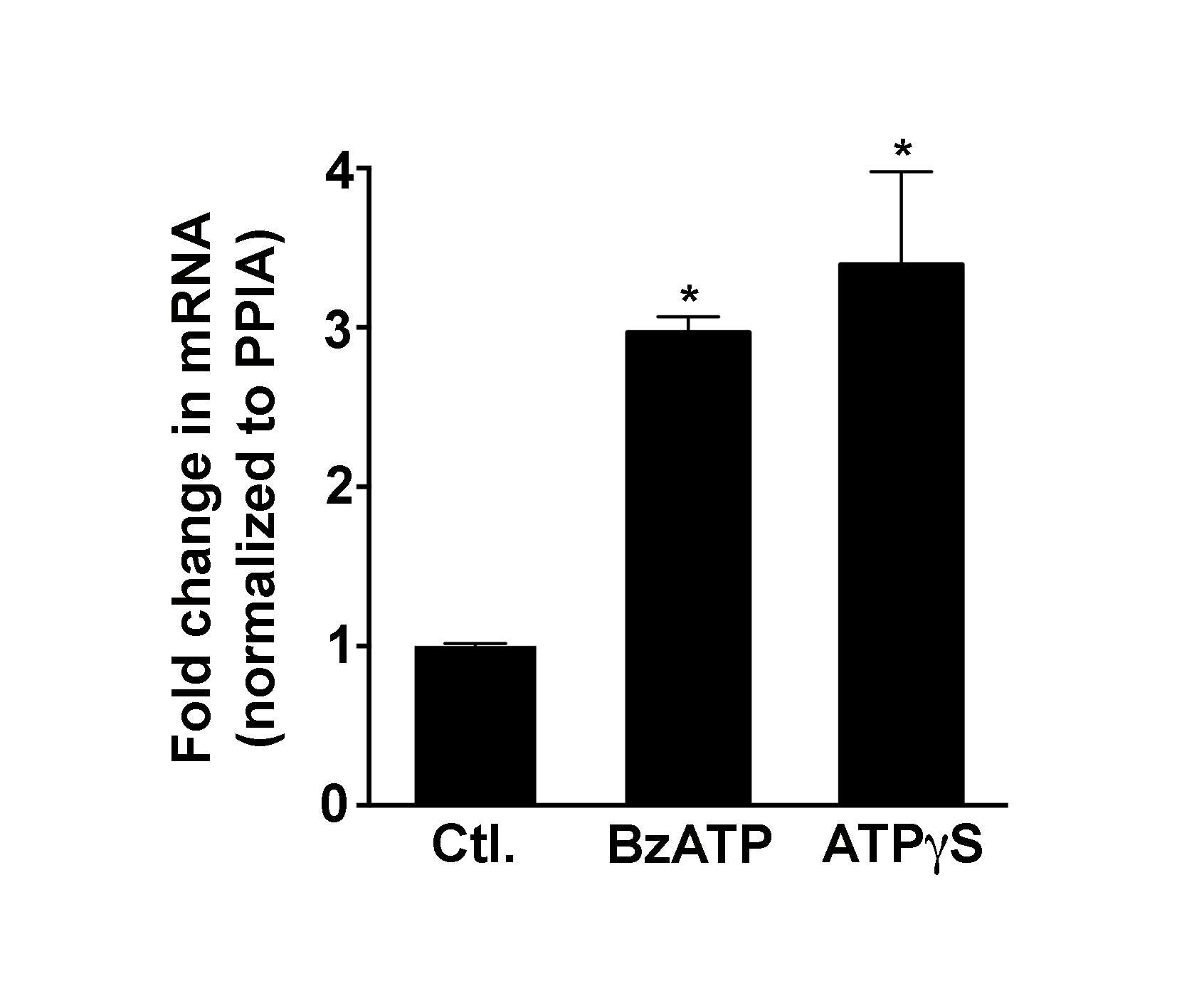

Supplement: S3 Fig — BzATP and ATPγS, the P2X7 agonists increase transcript levels (24 h) of IL-1β. Transcripts were normalized to the housekeeping gene, PPIA. n = 3 independent experiments each done in replicates; *p ≤ 0.05. (TIF) [file pone.0125111.s003.tif]
