# Supplementary material for: The ATP Receptors P2X7 and P2X4 Modulate High Glucose and Palmitate-Induced Inflammatory Responses in Endothelial Cells
Source: PLoS One. 2015 May 4;10(5):e0125111. doi: 10.1371/journal.pone.0125111 (PMC4418812; doi:10.1371/journal.pone.0125111)
Supplement: S1 Table — qRT-PCR analysis show fold change (relative to vehicle control) in high glucose and palmitate-induced (24 h) gene expression of P2 receptors, cytokines, chemokines, cyclooxygenase, mediators of inflammasome, and adhesion molecules normalized to the housekeeping gene (PPIA). n = 5 to 9 independent experiments each in replicates; p ≤ 0.05. (DOCX) [file pone.0125111.s004.docx]

| **Genes** | **qRT-PCR expression (Fold Change)** |
| --- | --- |
| **P2 Receptors** |  |
| *P2X4* | 3.6 ± 0.19; *p* = 0.002 |
| *P2X7* | 3.3 ± 0.35; *p* = 0.002 |
| **Cytokines, Chemokines, & Cyclooxygenase** |  |
| *IL-1β* | 6.33 ± 1.06; *p* < 0.0001 |
| *IL-6* | 38.25 ± 2.18; *p* < 0.0001 |
| *IL-8* | 22.44 ± 1.26; *p* < 0.0001 |
| *PTGS2* | 12.83 ± 0.68; *p* < 0.0001 |
| **Inflammasomes** |  |
| *CASP1* | 1.4 ± 0.04; *p* < 0.0001 |
| *CASP5* | 21 ± 2.7; *p* = 0.008 |
| *TXNIP* | 4.4 ± 0.46; *p* = 0.002 |
| **Adhesion Molecules** |  |
| *ICAM-1* | 47.8 ± 2.9; *p* < 0.0001 |
| *VCAM-1* | 11.4 ± 2.05; *p* < 0.0001 |
