# Supplementary material for: The ATP Receptors P2X7 and P2X4 Modulate High Glucose and Palmitate-Induced Inflammatory Responses in Endothelial Cells
Source: PLoS One. 2015 May 4;10(5):e0125111. doi: 10.1371/journal.pone.0125111 (PMC4418812; doi:10.1371/journal.pone.0125111)
Supplement: S2 Table — qRT-PCR analysis show fold change in response to 30 mmol/L mannitol (24 h; relative to vehicle control) in gene expression normalized to housekeeping gene (PPIA). n = 3 independent experiments each done in replicates. (DOCX) [file pone.0125111.s005.docx]

| **Genes** | **qRT-PCR expression (Fold Change)** |
| --- | --- |
| *P2X7* | 1.044 ± 0.03 |
| *VCAM-1* | 0.85 ± 0.03 |
| *ICAM-1* | 1.11 ± 0.12 |
| *IL-8* | 0.94 ± 0.07 |
| *PTGS2* | 1.09 ± 0.08 |
